# Baseline characteristics and seven-year follow-up of patients with coronary slow flow: A cohort study in northeastern Iran

**DOI:** 10.34172/jcvtr.33167

**Published:** 2025-03-18

**Authors:** Farima Farsi, Negar Morovatdar, Ali Eshraghi

**Affiliations:** ^1^Obesity and Eating Habits Research Center, Endocrinology and Metabolism Clinical Sciences Institute, Tehran University of Medical Sciences, Tehran, Iran; ^2^Clinical Research Development Unit, Faculty of Medicine, Mashhad University of Medical Sciences, Mashhad, Iran; ^3^Department of Cardiology, Faculty of Medicine, Mashhad University of Medical Sciences, Mashhad, Iran

**Keywords:** Adverse cardiac events, Coronary slow flow, Dyslipidemia

## Abstract

**Introduction:**

An angiographic finding known as "coronary slow flow phenomenon" (CSFP) occurs when there is no discernible stenosis but the contrast flow is slower than usual. Although the prognosis for the majority of CSFP cases is favorable, frequent angina significantly lowers their quality of life. Therefore, this study aimed to explore the potential contributing risk factors and prognostic implications of CSFP on long-term cardiovascular outcomes.

**Methods:**

This retrospective, cohort study was conducted between years 2014-2022 and included a total of 65 CSFP patients and 65 controls with normal coronary flow, as evidenced by coronary angiography. These two groups were examined in terms of future cardiovascular consequences due to this phenomenon, baseline demographic characteristics, and laboratory findings. A *P* value<0.05 was considered significant.

**Results:**

In this study 130 people including 73 men and 57 women, who because of the typical chest pain and at least a noninvasive test took angiography, were explored. The median triglyceride (200.80±48.51 vs 131.79±34.22, *P*<0.001), total cholesterol (189.46±10.84 vs 103.43±8.13, *P*<0.001), and low-density lipoprotein (153.28±34.28 vs 103.34±19.70, *P*=0.01) were significantly higher in the affected people. During clinical follow-up, a higher number of major adverse cardiac events (8.97±2.95 vs 4.52±2.12, *P*<0.001) was observed in the CSFP cases. Moreover, a one-unit increase in body mass index raised the probability of adverse cardiac events by 0.912 in CSFP cases.

**Conclusion:**

Our research indicated that individuals with CSFP were more likely to develop cardiac events including unstable angina. Furthermore, obesity and dyslipidemia could provoke this phenomenon.

## Introduction

 The term “coronary slow flow phenomenon” (CSFP) or syndrome Y refers to a condition where coronary angiography shows no significant lesion in the epicardial coronary arteries but with delayed blood perfusion. It is not associated with thrombolytic treatment, coronary angioplasty, arterial spasm, coronary dilatation or stenosis, cardiomyopathy, significant valvular heart disease, decompensated heart failure, and specific connective tissue disorders of the coronary microvasculature.^1^ Among patients who underwent coronary angiography due to chest pain, 1%-7% of them have confirmed CSFP. Additionally, four percent of patients with unstable angina also had CSFP. Clinical signs and symptoms of coronary atherosclerotic disease are comparable in CSFP.^2,3^ Repetitive typical chest pain is the most common symptom observed in patients with CSFP.^4^ Although the prognosis for the majority of CSFP cases is favorable,^5^ frequent angina significantly lowers the quality of life. Furthermore, this phenomenon could result in recurrent hospitalization, repeated coronary angiography, myocardial ischemia, and ultimately acute coronary syndrome (ACS). Young male patients and smokers are more likely to experience this condition.^3,6,7^ Other risk factors such as hypertension (HTN),^8^, low levels of high-density lipoprotein cholesterol (HDL-C),^9^ diabetes mellitus (DM), and high body mass index (BMI) have been suggested to correlate with this condition.^10^ The exact mechanism of CSFP is still unknown, however, its pathophysiology has been linked to several variables including microvascular abnormalities, inflammation, structural, and functional abnormalities of blood cells, vascular endothelial dysfunction, atherosclerosis, and metabolic disorders.^11^ Patients who were histologically investigated had the following findings: myofibrillar hypertrophy, inflammation of the wall of microvessels, endothelial damage, and arterial stenosis.^3,12^ CSFP currently lacks a definitive treatment due to its unclear mechanism. In the clinic, empirical treatment mostly consists of the following elements: management of cardiovascular risk factors, such as diabetes, hyperlipidemia, and hypertension,^13^ antiplatelet medications,^14^ calcium channel blockers, nitrate medications,^13^ and beta blockers.^15^

 One of the diagnostic methods is the stress exercise test, however, it could not detect all patients with CSFP.^16^ Another way to recognize this phenomenon is coronary angiography, which can determine the blood flow rate in myocardial arteries quantitatively and has been used extensively to assess acute myocardial infarction.

 Diagnosis of the cardiac source of chest pain in patients affected by CSFP has been challenging for cardiologists since their coronary angiography is nearly normal. Due to the unclear mechanism of this phenomenon, there is no definite cure, nor has the syndrome been extensively studied whether this angiographic finding is related to a pathological process in the coronary artery with an unfavorable prognosis for cardiovascular events or is natural. Considering that the most common manifestation of this phenomenon is frequent chest pain, it could have a significant impact on a person’s quality of life; therefore, we aimed to follow up patients who were referred to our clinic due to typical chest pain, underwent coronary angiography, and were diagnosed with CSFP. Furthermore, we examined the function of clinical traits in CSFP patients and served as a guide for future research into the possible mechanisms underlying CSFP.

## Materials and Methods

###  Coronary angiography

 Y syndrome is only diagnosed in patients whose angiography is relatively normal and is not influenced by atherosclerotic coronary artery disease. Intravascular ultrasound revealed that a large number of people whose angiogram was normal had extensive atherosclerotic heart disease.^17^ The researchers use two approaches to define the slow flow of contrast matter in angiography:

a) Thrombolysis in myocardial infarction (TIMI) flow grade: It is a semi-quantitative method that grades contrast material flow in epicardial vessels from TIMI-0 (no flow) to TIMI-3 (natural flow). TIMI-2 is considered to be turbidity at the end of the vascular bed. This method has been also used to detect the non-flow phenomenon.^18^
b) TIMI frame count: This approach was first devised by Gibson et al^19^, and is a quantitative method in which the number of frames required for the opacification of the vascular bed is calculated. The reference value of 23 ± 3 frames is considered, so some researchers estimate values higher than that to be slow flow ^17^ while others take into account the required number of frames greater than two standard deviations of reference value as CSFP.^7^ It is worth noting that studies using this method have reported an average of more than 50 frames as CSFP.^18^

###  Baseline definition and measurements

 In the current study, having a fasting blood sugar (FBS) of at least 126 mg/dl twice in addition to clinical symptoms (weight loss, polyuria, and polydipsia), a Hemoglobin A1C (HbA1C) of at least 6.4, or a prior history of the illness were all considered indicators of DM. A normal lipid profile was defined as low-density lipoprotein (LDL-C) < 100 mg/dl, HDL-C ≥ 50 mg/dl for women HDL-C ≥ 40 mg/dl for men, and total cholesterol < 200 mg/dl. Hypertension was described as systolic blood pressure (SBP) ≥ 140 mmHg and/or diastolic blood pressure (DBP) ≥ 90 mmHg.

 Based on the angiographic findings of the patients, CSFP was defined as delayed distal opacification (TIMI-flow 2) in at least one epicardial vessel (Table 1). For additional insight into the coronary angiographic findings, please refer to Supplementary files 1-4.

**Table 1:** Proposed criteria for defining CSFP

**Angiographic evidence of the CSFP, defined by:**	**Exclusion of secondary causes of the CSFP, including:**
• No evidence of obstructive epicardial CAD (i.e. No angiographic lesions ≥ 40%)	• No reflow phenomenon
• Delayed distal vessel contrast opacification as evidenced by either:	• Coronary emboli
a. TIMI-2 flow (i.e. requiring ≥ 3 beats to opacify the vessel), or	• Coronary ectasia
b. Corrected TIMI frame count ≥ 27 frames (images acquires < 30 frames/s)	• Exogenous vasoconstrictor administration (e.g., cocaine)

Abbreviations: CSFP, coronary slow flow phenomenon; CAD, coronary artery disease; TIMI, thrombolysis in myocardial infarction. Criteria adapted from Nurkalem et al. (2008).^12^

###  Design and population of the study

**Figure 1:**
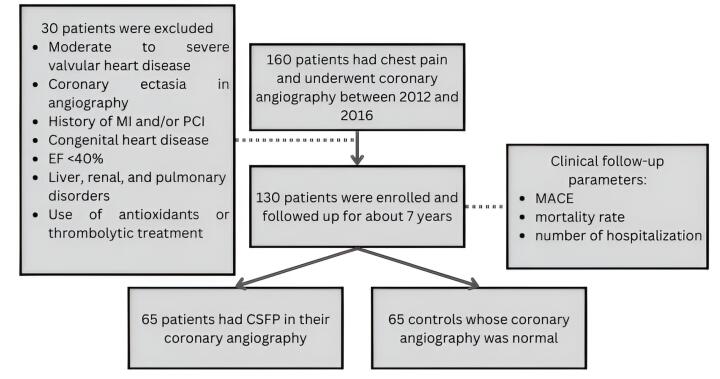
Flowchart of enrolment of the study participants, abbreviations: MI, myocardial infarction, PCI, percutaneous coronary intervention, EF, ejection fraction, MACE, major adverse cardiac events, CSFP, coronary slow flow phenomenon

 This was a seven-year cohort study, conducted in Imam Reza Teaching Hospital, a referral hospital in Iran. The protocol of the study was approved by the research ethics committee of Mashhad University of Medical Sciences (ethical code: IR.MUMS.MEDICAL.REC.1401.086). In total, 65 consecutive patients with CSFP and 65 normal controls were enrolled between March 2012 and February 2016 and were followed for 84 months ± 14 days (Figure 1). The exclusion criteria were as follows: moderate to severe valvular heart disease, heart failure (ejection fraction less than 40%), acute or chronic inflammatory disorders, renal disorders (protein-losing nephropathy with Albumin-creatinine ratio (ACR) < 300 and/or glomerular filtration rate (GFR) < 90 ml/min), liver disorders, coronary ectasia in angiography, history of myocardial infarction (MI) and/or primary cutaneous intervention (PCI), non-sinus rhythm in electrocardiogram (ECG), congenital heart disease, infection or having fever, chronic obstructive pulmonary disease (COPD), antioxidants consumption, and thrombolytic treatment. All patients provided informed consent and the study was conducted in accordance with the ethical principles described by the declaration of Helsinki.

###  Follow-up

 In this retrospective cohort study 130 patients who had typical chest pain and a positive result of a non-invasive test such as an exercise stress test or an indication for performing angiography including a strong family history of cardiovascular problems, old age or marked functional limitation, etc., and underwent angiography between March 2012 and February 2016 were enrolled and followed up for 84 months ± 14 days by maintaining consistent communication every year to prevent loss of communication. These subjects were followed for seven years for mortality rate, number of hospitalizations, and major adverse cardiac event (MACE, need for or performing repeated interventions such as PCI and coronary artery bypass graft, acute coronary syndrome) which were performed while explaining the study and assuring appropriate use of the information. Furthermore, demographic characteristics, angiographic findings, and laboratory parameters were explored using their medical records at the hospital and physical examination performed on arrival.

###  Statistical analysis

 The final data were analyzed using IBM SPSS Statistics for Windows, Version 23 (IBM Corp., Armonk, NY, USA). The data were described using the statistical indices mean, frequency, and standard deviation. Depending on the normality of data distribution the Student’s t-test was used to investigate the quantitative variables, while the qualitative variables were analyzed using the chi-squared test. To determine the relationship between MetS and the variables, we used Logistic Regression. The significance level was considered as *P* < 0.05 for all the comparisons.

## Results

 In this retrospective cohort study, 130 patients were enrolled and clinically followed up for 84 months ± 14 days. Among the studied population, sixty-five cases had CSFP in their angiograms, and an equal number did not. Of these, 73 were men (56.15%) and 57 were women (43.85%).

###  Demographic indices in two groups with CSFP and normal group

 According to Table 2, the number of diabetics, people with abnormal lipid profile and mean BMI in the CSFP group were 67.69% (vs. 47.69%, *P* = 0.03), 52.31% (vs. 47.69%, *P* = 0.03), and 27.18 ± 1.18 (vs 24.98 ± 1.44, *P* > 0.001), respectively. There was no significant difference in age (*P* = 0.47), gender (*P* = 0.72), smoking (*P* = 0.59), and systolic blood pressure (*P* = 0.55).

**Table 2:** Comparison of demographic indices between two groups with and without CSFP

**Demographic characteristics**	**Total** **(n=130)**	**CSFP group** **(n=65)**	**Normal group** **(n=65)**	***P*** ** value**
Age (years)	63.18 ± 11.07^a^	62.26 ± 10.68^a^	64.09 ± 11.45^a^	0.47
Gender				0.72
Female	57 (43.85%)	27 (41.53%)	30 (46.15%)	
Male	73 (56.15%)	38 (58.46%)	35 (53.84%)	
SBP (mmHg)	140.88 ± 9.96^a^	141.4 ± 8.02^a^	140.37 ± 11.63^a^	0.55
DM	75 (57.7%)	44 (67.69%)	31 (47.69%)	< 0.03
Smoking	48 (36.92%)	22 (33.85%)	26 (40.00%)	0.59
Abnormal lipid profile	65 (50%)	34 (52.31%)	31 (47.69%)	0.03
BMI	26.08 ± 1.71^a^	27.18 ± 1.18^a^	24.98 ± 1.44^a^	< 0.001

Values are counts (percentages) unless stated otherwise, ^a^ Mean ± standard deviation. Percentages are estimated within each group with and without CSFP. Abbreviations: CSFP, coronary slow flow phenomenon, SBP, systolic blood pressure, DM, diabetes mellitus, BMI, body mass index.

###  Laboratory data in patients with and without CSFP

 In terms of laboratory parameters, mean monocyte count, hemoglobin, blood glucose, total cholesterol, triglyceride, and LDL-C in the CSFP group were 0.39 ± 0.08 (vs. 0.33 ± 0.05, *P* = 0.01), 13.36 ± 1.96 (vs. 12.69 ± 1.72, *P* = 0.04), 103.69 ± 7.12 (vs. 90.43 ± 5.51, *P* > 0.001), 10.69 ± 10.64 (vs. 103.43 ± 8.13 vs. *P* > 0.001), 200.80 ± 48.51 (vs. 131.79 ± 34.22, *P* > 0.001 and 153.28 ± 34.28 (vs. 103.34 ± 19.70, *P* = 0.01) (Table 3). All of the CSFP patients had significantly higher amount of BMI, blood sugar, and total cholesterol than the normal group.

**Table 3:** Comparison of laboratory parameters between two groups with and without CSFP

**Laboratory data **	**Total (n=130)**	**CSFP group (n=65)**	**Normal group (n=65)**	***P*** ** value**
WBC count (10^9^/L)	8.18 ± 1.56	8.04 ± 1.54	8.37 ± 1.57	0.32
Monocyte count (10^9^/L)	0.36 ± 0.13	0.39 ± 0.08	0.33 ± 0.05	0.01
RBC count (10^9^/L)	4.58 ± 0.35	4.63 ± 0.37	4.52 ± 0.33	0.76
Hb (g/dl)	13.03 ± 1.87	13.36 ± 1.96	12.69 ± 1.72	0.04
RDW (%)	12.23 ± 1.63	12.01 ± 1.63	12.44 ± 1.62	0.13
BS (mg/dl)	97.06 ± 9.19	103.69 ± 7.12	90.43 ± 5.51	< 0.001
Total cholesterol (mg/dl)	146.45 ± 44.20	189.46 ± 10.64	103.43 ± 8.13	< 0.001
HDL-C (mg/dl)	47.22 ± 6.39	42.53 ± 6.56	51.91 ± 6.23	0.76
LDL-C (mg/dl)	128.31 ± 26.99	153.28 ± 34.28	103.34 ± 19.70	0.01
TG (mg/dl)	166.30 ± 41.37	200.80 ± 48.51	131.79 ± 34.22	< 0.001
ALT (IU/L)	25.82 ± 9.36	27.32 ± 10.11	24.10 ± 8.36	0.06
AST (IU/L)	30.92 ± 10.96	31.72 ± 11.50	30.12 ± 10.41	0.40
Creatinine (mg/dl)	1.09 ± 0.36	1.11 ± 0.36	1.05 ± 0.36	0.33

Values are Mean ± standard deviation. Abbreviations: CSFP, coronary slow flow phenomenon, WBC, white blood cell, RBC, red blood cell, Hb, hemoglobin, RDW, red cell distribution width, BS, blood sugar, HDL-C, high-density lipoprotein cholesterol, LDL-C, low-density lipoprotein cholesterol, TG, triglycerides, ALT, alanine aminotransferase, AST, aspartate aminotransferase.

 On binary logistic regression analysis on all subjects (Table 4), we observed that the higher BMI of the patient was most significantly associated with the occurrence of CSFP (odds ratio [OR] = 6.680 [3.250-13.730], *P* < 0.001). In addition to that, high BS (OR = 1.736 [1.412-2.135], *P* < 0.001), abnormal lipid profile (OR = 2.949 [1.440-6.038], *P* = 0.003), high total cholesterol (OR = 2.442 [1.853-3.031], *P* < 0.001, increased LDL-C (OR = 1.541 [1.012-2.070], *P* < 0.001), and TG (OR = 1.328 [1.006-1.650], *P* = 0.043) were associated with higher odds of CSFP.

**Table 4:** Binary logistic regression analysis for prediction of CSFP

**Variable**	**OR**	**95% CI for EXP (B)**		***P*** ** value**
		**Lower**	**Upper**	
DM	0.435	0.213	0.887	0.022
Abnormal lipid profile	2.949	1.440	6.038	0.003
BMI	6.680	3.250	13.730	< 0.001
Monocyte count	0.573	0.130	0.998	0.064
Hb	0.876	0.143	1.609	0.544
BS	1.736	1.412	2.135	< 0.001
Total cholesterol	2.442	1.853	3.031	< 0.001
LDL-C	1.541	1.012	2.070	< 0.001
TG	1.328	1.006	1.650	0.043

Abbreviation: DM, diabetes mellitus, BMI, body mass index, Hb, hemoglobin, BS, blood sugar, LDL-C, low-density lipoprotein cholesterol, TG, triglycerides.

###  Follow-up data of the two groups with and without CSFP

 As shown in Table 5, the mean number of hospitalizations, ACS, stable angina, and MACE in the CSFP group was 1.50 ± 2.05 (vs. 1.10 ± 1.89 and *P* = 0.03), 1.75 ± 1.2 (vs. 0.89 ± 0.95 and *P* > 0.001), 0.93 ± 5.45 (vs. 0.78 ± 2.78 and *P* > 0.001), 2.95 ± 8.97 (vs. 4.52 ± 2.12 vs. *P* > 0.001), respectively. The number of deaths during these seven years of follow-up war 6 in CSFP patients (vs. 0, *P* = 0.03), which was not cancer- or trauma-related. Furthermore, the CSFP cases had lower performance levels than the normal group (*P* < 0.001).

**Table 5:** Seven-year follow-up data of the two groups with and without CSFP

**Follow-up data**	**Total** **(n=130)**	**CSFP group** **(n=65)**	**Normal group** **(n=65)**	***P*** ** value**
Hospitalization	1.96 ± 1.33	2.05 ± 1.50	1.89 ± 1.10	0.03
MACE	6.75 ± 3.40	8.97 ± 2.95	4.52 ± 2.12	< 0.001
Death	6^a^	6^a^	0^a^	0.03
ACS	1.35 ± 1.13	1.75 ± 1.20	0.95 ± 0.89	< 0.001
Stable angina	4.12 ± 1.59	5.45 ± 0.93	2.78 ± 0.78	< 0.001
NYHA functional class				< 0.001
Class I		5 (7.69%)^a^	30 (46.15%)^a^	
Class II		37 (59.92%)^a^	35 (53.85%)^a^	
Class III		17 (26.15%)^a^	0 (0.00%)^a^	
Class IV		6 (9.23%)^a^	0 (0.00%)^a^	

Values are Mean ± standard deviation unless stated otherwise, ^a^ number (percentage). Abbreviations: CSFP, coronary slow flow phenomenon, MACE, major adverse cardiac events, ACS, acute coronary syndrome, NYHA, New York Heart Association.

**Figure 2:**
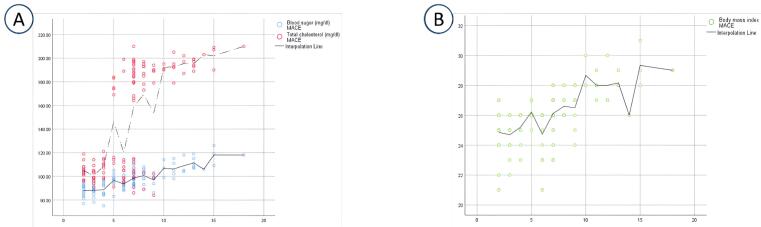
Correlation of Blood sugar, total cholesterol (A), body mass index (B), and major adverse cardiac events in the study population

 As illustrated in Figure 2, by increasing blood glucose, body mass index, and total cholesterol, MACE level increases in a relatively linear manner. Linear regression test was used to create a model that could exactly determine a linear relationship between MACE as the dependent variable and BS, total cholesterol, and BMI. A one-unit increase in BS, total cholesterol, and BMI raised the MACE rate by 0.293 (0.293 × BS-21.731 = MACE, *P* < 0.001), 0.053 (0.053 × total cholesterol-0.930 = MACE, * P* < 0.001), and 0.912 (0.912 × BMI-17.055 = MACE, *P* < 0.001), respectively.

## Discussion

 CSFP is a relatively common finding in angiograms of people who undergo angiography due to cardiac chest pain and a disruptive result of a noninvasive test.

 In the study of Wang et al CSFP had similar clinical manifestations of coronary atherosclerosis disease, which is consistent with our findings. Based on our study, such as that of John Beltreem et al,^6^ the MACE rate and the frequency of hospitalization and deaths related to or without cardiovascular disorders were significantly higher in the CSFP group, which contradicted the results of Mosseri et al ^3^ and Sadamusta et al,^20^ in which it was noted that this phenomenon had a good prognosis, however, due to the association of this phenomenon with adverse cardiovascular events, the necessity of clinical follow-up of these patients was emphasized. In a study conducted by Sadr Ameli et al the most common complaint of patients with CSFP was recurrent non-typical chest pain. In their study, a significant number of patients required re-angiography, and one-sixth of them had significant narrowing in their coronary arteries. ^21^ According to our study, a significant number of patients reported a history consistent with acute coronary syndrome. Mosseri et al^3^ discovered that recurrent chest pain was the most typical way that this phenomenon manifested itself, and the majority of patients in our study had a history of recurrent cardiac chest pain. In the study of Alvarez et al it was also noted that CSFP should be considered as a separate differential diagnosis for acute chest pain.^22^ The level of performance of the CSFP cases according to NYHA classification was in higher stages than the normal group.

 In this study, the risk factors that could according to the previous studies predict the occurrence of this phenomenon were also evaluated. Wang et al concluded that CSFP was more common in males and smokers ^2^, and Weferling et al noted in their research that the only difference in basic characteristics of those with and without this phenomenon was smoking ^23^ but in our study there was no significant relationship between smoking and male gender and this phenomenon. In the study of Huang et al and Afsin et al it has been suggested that metabolic syndrome and atherosclerotic diseases could be predictors of this phenomenon.^11,24^ According to our study, the number of people with diabetes, abnormal lipid profile, and the mean BMI in the CSFP group were significantly higher than the normal group. Furthermore, Zavala-Alarcon et al also and Seyyed Mohammadzad et al mentioned high BMI and diabetes as risk factors for this phenomenon.^10,25^ In the study of Pekdemir et al disseminated calcification, and atherosclerosis were also proposed as the underlying mechanism of this phenomenon.^26^ Moreover, Binak et al found a significant relationship between blood sugar levels and susceptibility to this phenomenon.^27^ There was no significant difference in age (*P* value = 0.479), gender (*P* value = 0.72), smoking (*P* value = 0.59), and systolic blood pressure (*P* = 0.55). Unlike the study of Alarcon et al and Sezgin et al which identified high blood pressure and low HDL-c as predisposing factors to this syndrome,^10,28^ in our study there was no significant relationship between systolic blood pressure and HDL cholesterol and this phenomenon. In another study performed on CSFP patients and control group, there was no difference in blood pressure level.^29^

 In the study conducted by Hockey Simsek et al,^15^ the use of Nebivolol (a Beta-blocker) was found to be effective in preventing from this phenomenon, especially from related arrhythmias, but in our study no difference was observed in the use of beta-receptor blockers between the CSFP and normal groups. Xia et al have suggested platelet inhibitors for coronary artery dilation,^14^ but accordingly in our study, its beneficial effect in preventing CSFP was not affected in any way by its use between the two groups with and without this phenomenon. Also, unlike the study that Zavala Alarcon and his colleagues had conducted on calcium receptor inhibitors and its effect on prevention of this phenomenon,^10^ its use did not differ significantly between the two groups in our study, and as a result, it had no detrimental effect on CSFP prevention.

 In terms of laboratory parameters, the mean monocyte count, hemoglobin, blood sugar, total cholesterol, triglyceride, and LDL-c in CSFP cases were higher than controls. There was no significant difference in the type and amount of medications used between the normal and CSFP subjects. Therefore, no specific treatment could be suggested that can be effective in preventing the occurrence of this phenomenon.

## Conclusion

 According to our study, this phenomenon could affect the quality of life and be a predictor of adverse cardiovascular events. Therefore, the need for clinical follow-up of patients to understanding the underlying pathophysiology of this phenomenon and providing suitable treatment is emphasized.

## Competing Interests

 The authors declare that there is no conflict of interest.

## Ethical Approval

 The research ethics committee at Mashhad University of Medical Sciences granted approval for the study protocol, assigned the ethical code IR.MUMS.MEDICAL.REC.1401.086. Written informed consent was obtained from the patients to publish this report in accordance with the journal’s patient consent policy.

## Supplementary Files


Supplementary file 1. Coronary Angiography - Pathologic Left Anterior Oblique (LAO) Cranial View Progression (CSFP observed)

Supplementary file 2. Coronary Angiography - Pathologic LAO Caudal View Progression (CSFP observed)

Supplementary file 3: Coronary Angiography - Normal LAO Cranial View

Supplementary file 4: Coronary Angiography - Normal LAO Caudal View
