# Supplementary figures and images for: Baseline characteristics and seven-year follow-up of patients with coronary slow flow: A cohort study in northeastern Iran
Source: J Cardiovasc Thorac Res. 2025 Mar 18;17(1):20–6. doi: 10.34172/jcvtr.33167 (PMC12068793; doi:10.34172/jcvtr.33167)

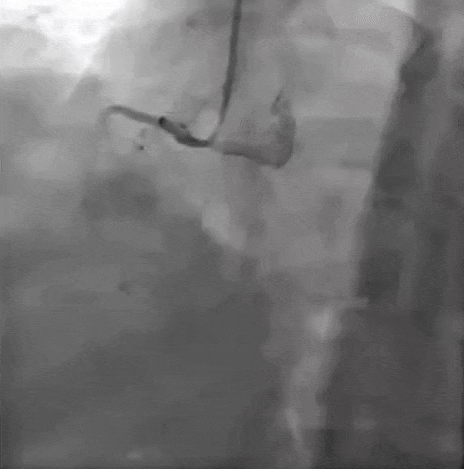

Supplement: Supplementary file 1 — Coronary Angiography - Pathologic Left Anterior Oblique (LAO) Cranial View Progression (CSFP observed) [file jcvtr-17-20-s001.gif]

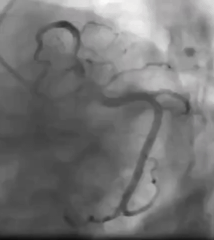

Supplement: Supplementary file 2 — Coronary Angiography - Pathologic LAO Caudal View Progression (CSFP observed) [file jcvtr-17-20-s002.gif]

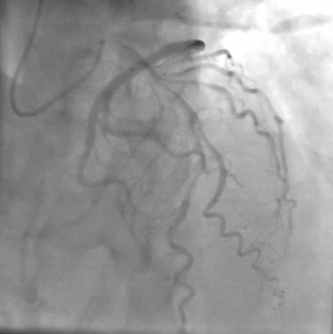

Supplement: Supplementary file 3 — Coronary Angiography - Normal LAO Cranial View [file jcvtr-17-20-s003.gif]

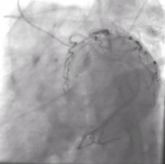

Supplement: Supplementary file 4 — Coronary Angiography - Normal LAO Caudal View [file jcvtr-17-20-s004.gif]
